# Formation of Zwitterionic Fullerodendron Using a New DBN-Focal Dendron

**DOI:** 10.3390/s100100613

**Published:** 2010-01-14

**Authors:** Yutaka Takaguchi, Maki Hosokawa, Masatoshi Mayahara, Tomoyuki Tajima, Takahiro Sasamori, Norihiro Tokitoh

**Affiliations:** 1 Graduate School of Environmental Science, Okayama University, Tsushima-Naka 3-1-1, Kita-Ku, Okayama 700-8530, Japan; E-Mails: gev19374@cc.okayama-u.ac.jp (M.H.); gev17371@cc.okayama-u.ac.jp (M.M.); tajimat@cc.okayama-u.ac.jp (T.T.); 2 Institute for Chemical Research, Kyoto University, Uji, Kyoto 611-0011, Japan; E-Mails: sasamori@boc.kuicr.kyoto-u.ac.jp (T.S.); tokitoh@boc.kuicr.kyoto-u.ac.jp (N.T.)

**Keywords:** dendrimer, fullerodendron, fullerene, DBN, fullerene anion

## Abstract

A new poly(amidoamine) dendron having 1,5-diazabicyclo[4.3.0]non-5-ene (DBN) at the focal point was synthesized. Interestingly, formation of zwitterionic fullerodendrons (λ_max_ = 930 nm for C_60_ and 795 nm for C_70_) were observed by Vis-NIR spectroscopy upon the reaction of C_60_ or C_70_ with the DBN-focal dendron. In particular, the C_70_ anion was effectively stabilized by the site isolation effect of the dendritic wedge. The half-life of fullerodendron **12b** having C_70_ anion at the focal point reaches 7,345 min, which is 20 times longer than that of complex between C_60_ and pristine DBN. Furthermore, in order to confirm the structure of the zwitterionic complex, fullerodendron **12a** was reprecipitated from benzonitrile/1,2,4-trimethylbenzene, and was observed using IR spectroscopy and APPI-MS.

## 1. Introduction

The sensing of fullerenes (C_60_, C_70_, and higher fullerenes) is gaining considerable interest because of their versatile applications in material science and nanotechnology, such as solar cells and field effect transistors (FETs). From this point of view, reversible formation of fullerodendrons is important [1–4], since the fullerodendron is known to be very soluble fullerene derivative. Meanwhile, Hirsch and co-workers reported that 1,8-diazabicyclo[5.4.0]undec-7-ene (DBU) reacts with C_60_ to give a zwitterionic complex via single electron transfer and radical recombination [5]. Recently, Nagata and coworkers have reported that this reaction is useful for large scale separation of C_60_ from a fullerene mixture because of selective complexation of higher fullerenes with DBU [6]. This zwitterion formation is known to be reversible reaction. However, the resulting complexes are susceptible to air-oxidation; they must therefore be handled in an inert atmosphere. Although many studies on the fullerodendron having neutral C_60_ or C_70_ moiety at the focal point have been described [7–29], there are few examples of incorporation of unstable fullerene species into dendritic architectures. Meanwhile, several groups have reported that covalent incorporation of highly unstable subunits into the dendritic architecture is effective to obtain active-site mimics for enzymes [30] and/or highly reactive species as an isolable compound [31] because of site isolation effect of the dendrimer [32–34]. In this context, site-isolation of dendritic substituent expected to be effective to stabilize the unstable fullerene species, such as fullerene anion. These backgrounds prompted us to investigate stabilization of the zwitterionic complex between fullerene and bicyclic amidines using the site isolation effect of the dendron. Herein we report the synthesis of a new DBN-focal dendron, poly (amidoamine) dendron having 1,5-diazabicyclo [4.3.0] non-5-ene (DBN) at the focal point together with the formation of zwitterionic fullerodendrons via complexation of fullerene, either C_60_ or C_70_, and DBN moiety of the dendron.

## 2. Results and Discussion

**Figure 1.**
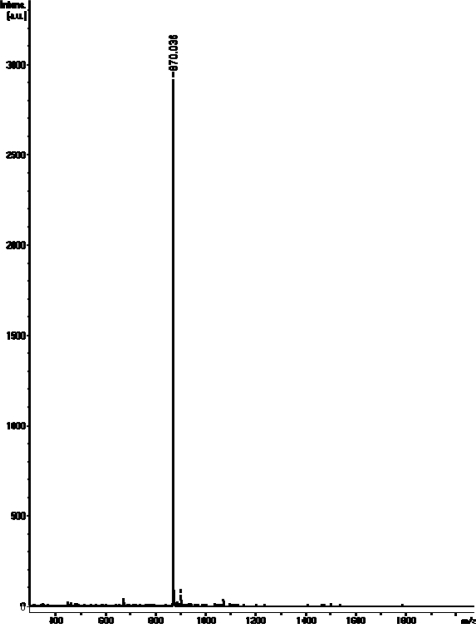
MALDI-TOF MS spectrum of dendron **1b**.

**Scheme 1.**
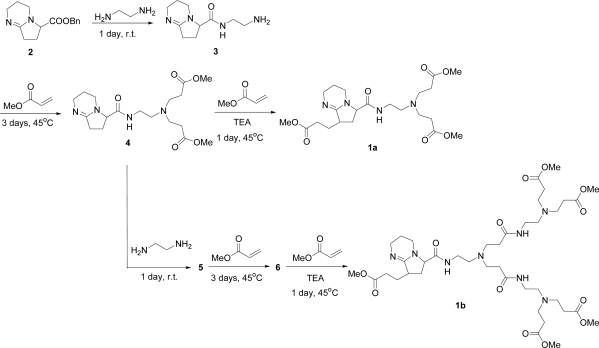
Syntheses of dendrons **1a** and **1b**.

DBN-focal poly(amidoamine) dendrons **1a** and **1b** were synthesized by the use of the divergent method shown in Scheme 1. A focal point of the dendron, 9-benzyloxycarbonyl-1,5-diazabicyclo [4.3.0] non-5-ene (**2**), was prepared as described by Kumagai *et al.* [35]. Compound **2** was allowed to react with ethylenediamine to afford DBN derivative **3**. Then, treatment of **3** with methyl acrylate produced dendron **4**. Subsequent reaction of **4** with methyl acrylate in the presence of triethylamine (TEA) produced dendron **1a** in 40% yield. This three-step process can be repeated to prepare dendron **1b** in 26% yield. The structures of dendrons **1a** and **1b** were confirmed by ^1^H- and ^13^C-NMR spectroscopies and MALDI-TOF-MS. In the ^1^H-NMR spectra of dendron **1a**, multiplet peaks around 4.9 ppm are attributable to the methine proton of DBN’s 9-position of diastereomeric mixture of dendron **1a**. Furthermore, a broad peak around 3.2 ppm, which represents the methine proton of DBN’s 7-position, was observed. In the ^13^C-NMR spectra of dendron **1a**, the chemical shift at δ 166.7 is the imine carbon signal of the focal point. The MALDI-TOF-MS spectrum of **1a** showed a molecular ion peak at *m/z* 469.27 (**1a**, C_22_H_37_N_4_O_7_ requires *m/z* 469.26) using positive-ion mode. Figure 1 shows that the MALDI-TOF-MS spectrum of **1b** exhibits a molecular ion peak at *m/z* 870.04 (**1b**, C_40_H_69_N_8_O_13_ requires *m/z* 869.49) using positive-ion mode.

**Scheme 2.**
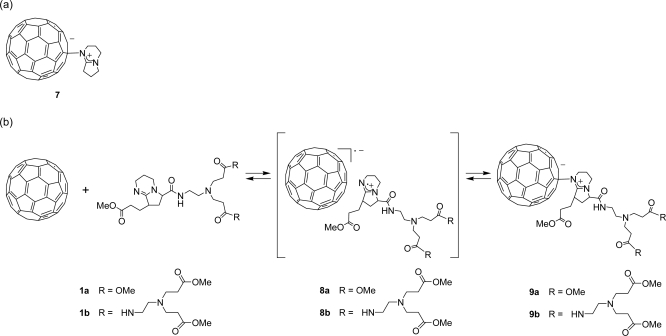
(a) Structure of zwitterionic complex **7**. (b) Formation of zwitterionic fullerodendrons **9a** or **9b**.

The formation of zwitterionic complex of dendrons **1a** or **1b** with C_60_ was observed by Vis/NIR spectrum, as reported by Hirsch *et al.* (Scheme 2) [5]. In a typical experiment, C_60_ (0.200 mg, 0.278 × 10^−3^ mmol) was dissolved in benzonitrile (3 mL), to which dendron **1b** (9.49 mg, 0.0109 mmol) was added under an Ar atmosphere. Subsequently, the Vis/NIR spectrum of the solution was recorded.

**Figure 2.**
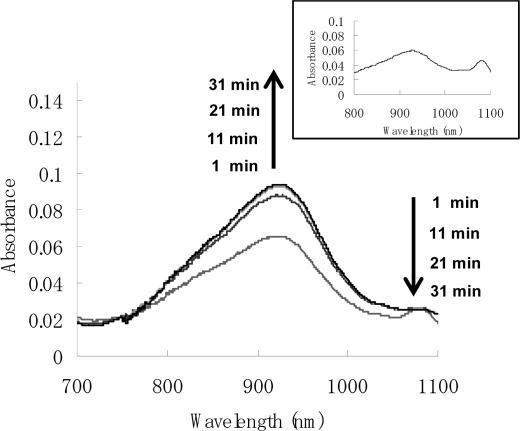
Vis-NIR spectra of the zwitterion **9b** and radical ion pair **8b** in benzonitrile. Inset: Vis-NIR spectrum of zwitterion **7**.

**Figure 3.**
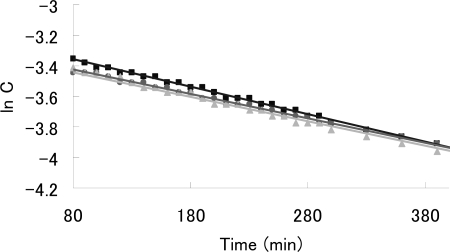
Time profile of the concentration of the zwitterions **7** (▪), **9a** (•), and **9b** (▴).

Figure 2 shows the Vis/NIR spectrum of the reaction of dendron **1b** with C_60_. The decay of the absorbance of C_60_ anion radical (λ_max_ = 1083 nm) is accompanied by the evolution of the zwitterion (λ_max_ = 930 nm) [5]. These assignments were confirmed by the reaction of C_60_ with pristine DBN, in which radical ion pair (λ_max_ = 1083 nm) and zwitterionic complex **7** (λ_max_ = 930 nm) were also observed. Although the zwitterionic fullerodendrons **9a** and **9b** were stable under an Ar atmosphere for 24 h at room temperature, they slowly decomposed on exposure to air, as reported by Nagata *et al.* [6]. We examined the time course of the absorbance of zwitterionic complexes **7**, **9a** and **9b** (λ_max_ = 930 nm) in the presence of atmospheric oxygen (Figure 3). The half-lives of the zwitterion complexes **7**, **9a** and **9b** were estimated as 377, 397 and 463 min, respectively. Although a clear difference of half-lives between zwitterions **7** and **9a** could not be found, the highest generation **9b**, which might be stabilized by the site isolation effect of dendritic wedge, showed the longest half-life.

**Figure 4.**
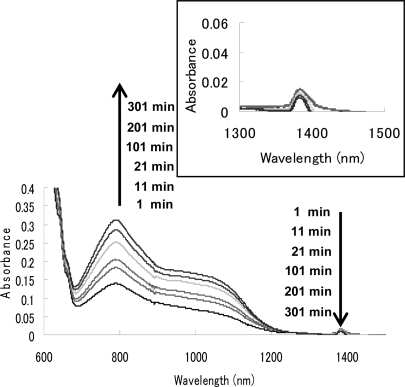
Vis-NIR spectra of the zwitterions **12b** and radical ion pair **11b** in benzonitrile. Inset: Expanded Vis-NIR spectrum of radical ion pair **11b**.

**Figure 5.**
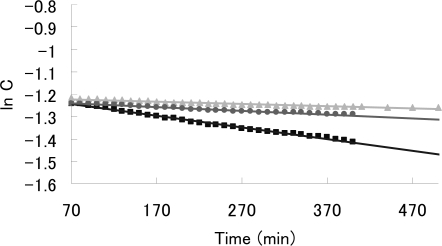
Time profile of the concentration of the zwitterions **10** (▪), **12a** (•), and **12b** (▴).

**Scheme 3.**
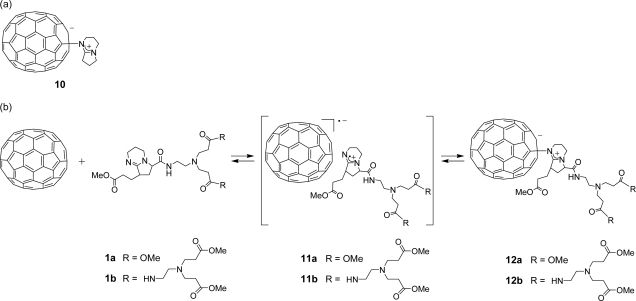
(a) Structure of zwitterionic complex **10**. (b) Formation of zwitterionic fullerodendrons **12a** or **12b**.

In order to obtain a more stable zwitterionic fullerodendron, we examined the respective reactions of dendrons **1a** and **1b** with C_70_, which has higher electron affinity than C_60_ (Scheme 3). In a typical experiment, C_70_ (0.220 mg, 0.262 × 10^−3^ mmol) was dissolved in benzonitrile (3 mL), to which dendron **1b** (9.49 mg, 0.0109 mmol) was added under an Ar atmosphere. Subsequently, the Vis/NIR spectrum of the solution was observed. Figure 4 shows the Vis/NIR spectrum of the reaction of dendron **1b** with C_70_. The decay of the absorbance at 1,380 nm is accompanied by the evolution of the absorbance at 795 nm. We interpreted that these absorbances were derived from a radical ion pair (λ_max_ = 1,380 nm) and zwitterionic complex (λ_max_ = 795 nm), as reported by Fukuzumi *et al.* [36]. These assignments were confirmed by the reaction of C_70_ with pristine DBN, in which a radical ion pair (λ_max_ = 1,380 nm) and zwitterionic complex **10** (λ_max_ = 795 nm) were also observed. Although the zwitterionic fullerodendrons **12a** and **12b** were stable under an Ar atmosphere for 5 days at room temperature, they decomposed slowly on exposure to air as well as in the case of C_60_. We examined the time course of the absorbance of zwitterionic complexes **10**, **12a** and **12b** (λ_max_ = 795 nm) in the presence of atmospheric oxygen (Figure 5). The half-lives of the zwitterion complexes **10**, **12a**, and **12b** were estimated as 1445, 4800 and 7345 min, respectively. The half-lives of zwitterions having dendritic wedge, compounds **12a** and **12b,** were longer than that of **10**. Comparing half-lives of fullerodendrons **12a** and **12b**, it is obvious that the stability of zwitterions depends on the generation of the dendron unit. This result indicated that zwitterionic complexes **12a** and **12b** might be stabilized by the site isolation effect of the dendritic wedge.

**Figure 6.**
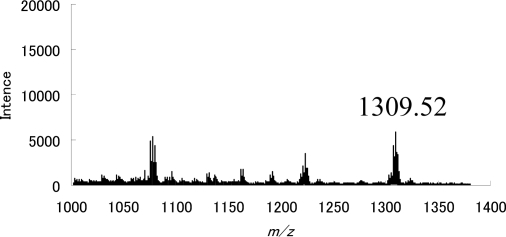
APPI MS spectrum of zwitterionic fullerodendron **12a**.

The absorption maxima and half-lives of zwitterions **7**, **9**, **10** and **12** were summarized in Table 1. Comparing the half-lives of zwitterionic complexes **7**, **9**, **10** and **12**, we can conclude that anionic fullerene moieties of zwitterionic fullerodendrons **9b** and **12b** are stabilized by the site isolation effect of the dendritic wedge. In particular, zwitterionic fullerodendron **12b**, which has a C_70_ moiety at the focal point, showed remarkable stability compared with **10**, which does not have a dendritic wedge, and fullerodendron **9b**, which contains C_60_ moiety at the focal point. These observed results might be due to higher electron affinity of C_70_ than C_60_, and the difference of anion delocalization between C_60_ and C_70_. In marked contrast with the complete anion delocalization of C_60_, the localized anion of C_70_ are known to be the reason of regioselective addition reactions [37]. Furthermore, the structure of zwitterionic fullerodendron **12a**, which could be isolated by reprecipitation from benzonitrile/1,2,4-trimethylbenzene, was confirmed by IR spectroscopy and APPI-MS. In the IR spectrum of fullerodendron **12a**, the –C = N absorbance (**1a**, 1,680 cm^−1^) of the DBN^+^ moiety is split into two bands at 1,663 and 1,674 cm^−1^. This splitting occurs because the two nitrogens in the DBN^+^ moiety are not identical; therefore two –C = N vibrations appeared as reported by Hirsch *et al.* [5]. The APPI-MS showed a molecular ion peak at *m/z* 1,309.52 (C_92_H_37_N_4_O_7_ requires *m/z* 1,309.27 [MH^+^]) and fragment peaks at 1,223.49 ([MH^+^]-CH_2_CH_2_COOCH_3_) and 1,077.52 ([MH^+^]-CH_2_CH_2_COOCH_3_-(CH_2_CH_2_COO)_2_) as shown in Figure 6.

## 3. Experimental Section

NMR spectra were measured using a spectrometer (AL 300; JEOL). Matrix-assisted laser desorption ionization time-of-flight mass spectroscopy (MALDI-TOF-MS) was performed on a mass spectrometer (Autoflex; Bruker Daltonics Inc.) using dithranol (1,8-dihydroxy-9-anthrone) as a matrix. Atmospheric pressure photo ionization mass spectroscopy (APPI-MS) was performed on a BRUKER micrOTOF focus-Kci mass spectrometer equipped with an APCI ionization unit. The GPC experiments (LC-918V; Japan Analytical Industry Co.) were performed using JAIGEL 1H, 2H (eluent: chloroform). The UV/vis spectra (λ_max_ in nm (ε)) were measured using a spectrophotometer (UV-3150, Shimadzu Corp.). Infrared (IR) spectra were measured using a spectrophotometer (Avatar 360T2: Thermo Nicolet). The reagents were obtained from Wako Pure Chemical Industries Ltd., Tokyo Kasei Kogyo Co. Ltd. Co., Aldrich Chemical Co. Inc., and Frontier Carbon Co. The reagents used as reaction solvents were further purified using general methods.

### Preparation of dendron **1a**

A suspension of 9-benzyl-1, 5-diazabicyclo [4.3.0] non-5-ene **2** (690 mg, 2.67 mmol) in methanol (18 mL) was added dropwise to a stirred solution of ethylenediamine (30.1 g, 534 mmol) in methanol (18 mL) at room temperature. The mixture was stirred continuously for 1 day. After removal of the solvent, the residue was washed with excess diethyl ether to obtain compound **3**, which was used for a following reaction without further purification. A mixture of **3** (530 mg, 1.88 mmol), methyl acrylate (3.24 g, 37.6 mmol), and methanol (50 mL) was stirred at 45 °C for 3 days. After removal of the solvent, the residue was purified using silica-gel column chromatography (eluent, chloroform/methanol = 15/1) to obtain compound **4**. Compound **4** (900 mg, 2.34 mmol), methyl acrylate (4.02 g, 46.8 mmol), triethylamine (0.240 g, 2.34 mmol), and methanol (63 mL) were stirred at 45 °C for 1 day. After removal of the solvent, the residue was purified by silica-gel column chromatography (eluent, chloroform/methanol = 15/1) to afford dendron **1a** (720 mg, 1.54 mmol) as a yellow oil in 40% yield: ^1^H-NMR (300 MHz, CDCl_3_) δ 2.18–2.24 (m, 3H), 2.43–2.47 (t, *J* = 6.6 Hz, 4H), 2.61–2.65 (dt, *J* = 4.8, 6.6 Hz, 2H), 2.75–2.79 (t, *J* = 6.6 Hz, 4H), 2.82–2.86 (m, 2H), 3.15–3.24 (m, 1H), 3.36–3.52 (m, 5H), 3.63–3.69 (m, 8H), 3.73 (s, 3H), 3.80–3.84 (t, *J* = 6.6 Hz, 2H), 4.87–4.92 (m, 1H), 8.10 (t, *J* = 4.8 Hz, 1H); ^13^C-NMR (CDCl_3_) δ 18.9, 24.0, 30.7, 31.6, 32.6, 37.4, 41.8, 44.7, 49.0, 51.9, 52.3, 52.5, 68.2, 77.2, 166.7, 168.7, 171.0, 173.1; IR (neat) ν_max_ = 1,664, 1,731 cm^−1^; MALDI-TOF Mass Found: *m/z* 469.27. Calcd. for C_22_H_37_N_4_O_7_: [MH^+^], 469.26.

### Preparation of dendron **1b**

A suspension of **4** (630 mg, 1.64 mmol) in methanol (11 mL) was added dropwise to a stirred solution of ethylenediamine (19.7 g, 328 mmol) in methanol (11 mL) at room temperature. The mixture was stirred continuously for 1 day. After removal of the solvent, the residue was washed with excess diethyl ether to obtain compound **5**, which was used for the following reaction without further purification. A mixture of **5** (560 mg, 1.28 mmol), methyl acrylate (4.40 g, 51.2 mmol), and methanol (69 mL) was stirred at 45 °C for 3 days. After removal of the solvent, the residue was purified using silica-gel column chromatography (eluent, chloroform/methanol = 10/1) to obtain compound **6**. A methanol solution (26 mL) of compound **6** (740 mg, 0.946 mmol), methyl acrylate (1.63 g, 19.0 mmol), and triethylamine (0.10 g, 0.989 mmol) was stirred at 45 °C for 1 day. After removal of the solvent, the residue was purified by silica-gel column chromatography (eluent, chloroform/methanol = 10/1) and GPC to afford the dendron **1b** (377 mg, 0.434 mmol) as a yellow oil in 26% yield: ^1^H-NMR (CDCl_3_) δ̣ 2.00–2.20 (m, 3H), 2.35 (t, *J* = 6.0 Hz, 8H), 2.44 (t, *J* = 6.3 Hz, 4H), 2.43–2.44 (m, 1H), 2.49–2.54 (dt, *J* = 5.4, 6.0 Hz, 4H), 2.60–2.65 (dt, *J* = 4.5, 6.0 Hz, 2H), 2.69 (t, *J* = 6.0 Hz, 8H), 2.76 (t, *J* = 6.3 Hz, 4H), 3.07–3.15 (m, 2H), 3.26–3.45 (m, 8H), 3.53–3.59 (m, 3H), 3.60 (s, 12H), 3.73 (s, 3H), 3.84 (t, *J* = 6.0 Hz, 2H), 4.87–4.93 (m, 1H), 7.13 (t, *J* = 5.4 Hz, 2H), 8.49 (t, *J* = 4.5 Hz, 1H); ^13^C-NMR (CDCl_3_) δ 18.5, 24.0, 25.6, 30.5, 32.7, 37.1, 37.6, 40.2, 41.9, 49.0, 49.2, 49.7, 50.8, 51.6, 52.3, 52.8, 67.6, 71.4, 166.5, 168.3, 171.0, 172.3, 173.1; IR (neat) ν_max_ = 1681, 1733 cm^−1^; MALDI-TOF Mass Found: *m/z* 870.04. Calcd. for C_40_H_69_N_8_O_13_: [MH^+^], 869.49.

### Formation of zwitterionic fullerodendrons **9** *or* **12**

A solution of dendron **1a** or **1b** (5.16 mg/9.56 mg, 0.0110 mmol) in benzonitrile (1.5 mL) was added to a solution of C_60_ or C_70_ (0.18 mM) in benzonitrile (1.5 mL). The solution was investigated using Vis/NIR.

### Preparation of zwitterionic fullerodendron **12a**

A solution of dendron **1a** (11.2 mg, 0.0239 mmol) in benzonitrile (2 mL) was added to a solution of C_70_ (1 mg) in 1,2,4-trimethylbenzene (5 mL). The mixture was stirred for 1 h at 25 °C under an Ar atmosphere. Centrifugation (4000 *g*) of the suspension for 30 min gave a brown precipitation: IR (neat) ν_max_ = 1,663, 1,674, 1,731 cm^−1^; APPI MS Found: *m/z* 1309.52. Calcd. for C_92_H_37_N_4_O_7_: [MH^+^], 1309.27.

## 4. Conclusions

The results described herein show the first example of a DBN-focal dendron and the formation of a zwitterionic fullerodendron, observed by UV-vis-NIR spectra, having an anionic fullerene moiety at the focal point. In particular, the C_70_ anion was effectively stabilized by the site isolation effect of the dendritic wedge. The lifetime of zwitterionic fullerodendron **12b** formed by the reaction of C_70_ with the DBN-focal dendron **1b** is approximately 20 times longer than that of zwitterionic complex between C_60_ and DBN. It is notable that the reversible formation of zwitterionic fullerodendrons potentially applicable to sensing fullerenes, because the absorption maximum of an anionic fullerene moiety should depend on the number of the carbon atoms in a fullerene. Further work is in progress to explore the selective sensing of the fullerene family using zwitterionic fullerodendrons.

## Figures and Tables

**Table 1.** Absorption maxima and half-lives of the zwitterionic complexes **7**–**12** in air.

**Compound**	**λ_max_ [nm]**	**half-life [min] ^a^**
**7**	930	377
**9a**	930	397
**9b**	930	463
**10**	795	1445
**12a**	795	4800
**12b**	795	7345

a Half-lives were estimated using pseudo-first order decays of the absorption spectra.
